# Bone Fillers with Balance Between Biocompatibility and Antimicrobial Properties

**DOI:** 10.3390/biomimetics10020100

**Published:** 2025-02-10

**Authors:** Bogdan Valeriu Sorca, Durmuş Alpaslan Kaya, Madalina Georgiana Albu Kaya, Marius Enachescu, Daniela-Madalina Ghetu, Laura-Bianca Enache, Iulian Boerasu, Alina Elena Coman, Laura Cristina Rusu, Rodica Constantinescu, Irina Titorencu

**Affiliations:** 1 Department of Oral Pathology, Multidisciplinary Center for Research, Evaluation, Diagnosis and Therapies in Oral Medicine, “Victor Babes” University of Medicine and Pharmacy Timisoara, 2 Eftimie Murgu Sq., 300041 Timisoara, Romania; bogdan.sorca@umft.ro (B.V.S.); laura.rusu@umft.ro (L.C.R.); 2 Department of Field Crops, Faculty of Agriculture, Hatay Mustafa Kemal University, Antakya-Hatay 31034, Turkey; dak1976@msn.com; 3 Collagen Department, INCDTP—Division Leather and Footwear Research Institute, 93 Ion Minulescu Str., 031215 Bucharest, Romania; coman.alina27@yahoo.com (A.E.C.); rodica.roxana@yahoo.com (R.C.); 4 Center for Surface Science and Nanotechnology, National University of Science and Technology Politehnica Bucharest, 313 Splaiul Independentei, 060042 Bucharest, Romania; marius.enachescu@cssnt-upb.ro (M.E.); laura.bianca@cssnt-upb.ro (L.-B.E.); iulian.boerasu@cssnt-upb.ro (I.B.); 5 Institute of Cellular Biology and Pathology “Nicolae Simionescu”, 8 B. P. Hasdeu Street, District 5, 050568 Bucharest, Romania; madalina.ghetu@icbp.ro (D.-M.G.); irina.titorencu@icbp.ro (I.T.)

**Keywords:** bone fillers, collagen, essential oil, biocompatibility, antimicrobial activity

## Abstract

Millions of people request bone regeneration every year, and the market for bone grafting materials has a positive trend. The most used biomaterials applied to replace and regenerate bone are based on collagen and different types of ceramics in order to mimic natural bone matrix. However, there are a lot of implant-associated infections after surgery, or the implants are rejected because of reduced biocompatibility, and this is why the research into graft bone materials is still a challenge. This study aims to develop and characterize novel biomimetic bone fillers which have simultaneously both antimicrobial properties and biocompatibility with human bone marrow—derived mesenchymal stem cells (BMSCs). Type I collagen and calcium triphosphate in a ratio of 1:1 were used as a control, according to our previous studies, and ZnO, functionalized with different percentages of *Satureja thymbra* L. essential oils, was added as an antimicrobial, promoting bone growth, mineralization, and formation. The bone fillers were obtained by freeze-drying in spongious forms and characterized by Fourier Transform Infrared Spectroscopy (FT-IR), Scanning Electron Microscopy (SEM), water uptake, biodegradability over time, antimicrobial activity against *Staphylococcus aureus* and *Escherichia coli* and viability and proliferation of human BMSCs. The graft material showed a higher porosity with interconnected pores, gradual resorption over time and a balance between antimicrobial properties and biocompatibility and was chosen as an ideal bone filler.

## 1. Introduction

Bone defects and losses as a result of trauma, infection, tumor resection and other bone diseases annually affect millions of people worldwide [1,2]. This is why there is an increasing prevalence of bone-related materials for defect filling on the medical market and it is still a research challenge [3,4,5,6]. Even though bone has the ability of self-regeneration, there are a lot of limitations due to large defects or infection of the tissues [7]. Allografts and autografts are still valid solutions, but they also have some limitations, such as the small amount compared to the number of patients and body rejection [8,9]. This is the main reason for continuous research in developing new bone grafts which mimic the composition, structure and biologic functionality of natural bone. It is well known that human bone consists of approximately 30% proteins and 70% inorganic apatite crystals (a type of organic ceramic) along with bone cells [6,10,11]. The predominant protein is type I fibrillar collagen (about 90%) and about 10% other proteins, such as fibronectin, osteopontin, and osteocentin [12,13,14]. Many researchers focused their studies on obtaining ideal scaffolds with a 3D porous structure, high porosity and interconnected pores to allow cell infiltration, to be biodegradable in a certain time but to have good mechanical properties at the same time, and to possess biocompatible and osteogenic properties [6,11,15,16,17,18]. Such materials were developed especially as bone fillers or grafts, which are developed for tissue regeneration or replacement in a natural way [19]. Having good osteoconductivity, biocompatibility, bioactivity, and biodegradability, calcium phosphate (CaP)-based ceramic proved to stimulate osteoblastic cells and to promote bone regeneration, being considered to have the most similar chemical composition to human bones [20,21,22]. Moreover, CaPs were used in combination with natural or synthetic polymers to improve mechanical and biocompatibility properties of biomaterials [8,23,24,25,26,27,28,29,30,31,32,33,34,35].

Researchers doped materials with Zn^2+^ cations in order to improve some biological properties, such as promoting cell proliferation, osteogenic activity, angiogenesis, inhibition osteoclast differentiation, and regulating the inflammatory reaction [36,37]. Zinc oxide (ZnO) is also known as a good antimicrobial agent primarily associated with the generation of reactive oxygen species (ROS), such as hydroxyl radicals and superoxide anions, and the release of Zn²⁺ ions. These free radicals cause oxidative stress and disrupt the integrity of the bacterial membranes, leading to impaired mitochondrial function, ultimately inducing bacterial cell death [38]. However, it has been demonstrated that ZnO at a concentration over 2% has cytotoxic effects. Recently, studies have shown that using a combination of ZnO and essential oil (EO) (*Salvia abrotanoides* oil) increases antioxidant properties [39]. A similar study on peppermint EO and ZnO nanoparticles showed antibacterial, antioxidant, hemostatic, anti-inflammatory, and wound-healing effects [40], as many other essential oils are used as medicinal remedies [41,42]. Both salvia and peppermint EOs with ZnO were used for skin wound healing. Bone graft or fillers cannot be efficient in infected bone without any antimicrobial agent, and antibiotics are being increasingly avoided because of bacterial resistance. The novelty of our studies is that we develop scaffolds based on collagen, β-tricalcium phosphate with a small amount of ZnO and different percentages of *Satureja thymbra* L. EOs in order to prevent or heal the infected bone.

*S. thymbra* L., a perennial species belonging to the Lamiaceae family, is naturally distributed across Mediterranean coastal areas [43]. Morphologically, *S. thymbra* L. is distinguished by its narrow, elongated leaves and aromatic properties, closely resembling those of *Thymus* species. Phytochemically, the plant is abundant in EOs, with γ-terpinene (39.23%), thymol (25.16%), p-cymene (7.17%) and carvacrol (4.18%) as the predominant compounds. These constituents impart significant antimicrobial and antioxidant properties to the plant, rendering it a valuable resource in ethnobotanical studies [44,45,46].

Traditionally, *S. thymbra* L. has been utilized in the treatment of digestive disorders and respiratory infections, including colds and throat inflammations. Notably, in the southern regions of Turkey, particularly in Hatay, *S. thymbra* L. is consumed as an infusion to mitigate symptoms associated with colds and throat infections.

Control of inflammation is indispensable for optimal bone regeneration. It is known that zinc plays a central role in the immune system. Moreover, it was demonstrated that bone implants with ZnO-nanoparticles have good antibacterial and osteogenic properties [47]. Also, *Satureja thymbra* essential oils have antimicrobial, antibiofilm and antioxidant properties [46,48]. Ghafarifarsani et al. presented that *Satureja* spp. essential oil is used to improve growth, hematological, immunological and antioxidant parameters and resistance to salinity stress [49].

The synergistic effect between the ZnO and *S. thymbra* L. EO arises from the combination of their mechanisms. The ROS generated by ZnO can intensify the oxidative damage caused to the bacterial cell [38], while the membrane-disruptive action of the EO can enhance the penetration of ZnO/Zn²⁺ ions into the bacterial cells [50,51,52]. This dual mode of action based on the oxidative stress combined with membrane destabilization increases the overall antibacterial activity beyond the effects of each component alone.

The aim of this study was to develop and characterize novel biomimetic bone fillers which have simultaneously both antimicrobial properties, because of the content of ZnO with *S. thymbra* L. EOs, and biocompatibility with human bone marrow-derived mesenchymal stem cells (BMSCs), due to collagen with calcium phosphate, which mimic the bone composition.

The spongious forms of collagen/CaP with ZnO with EOs were prepared by freeze-drying, and the obtained sponges were characterized by Fourier Transform Infrared Spectroscopy (FT-IR), water uptake, biodegradation and Scanning Electron Microscopy (SEM). In order to prove the efficiency of the obtained bone fillers, they were in vitro tested for antimicrobial activity and biocompatibility, and the balance between these properties was established.

## 2. Materials and Methods

### 2.1. Materials

The botanical material utilized in this study was sourced from the Medicinal and Aromatic Plants collection garden at Hatay Mustafa Kemal University. Following harvest, the plant materials were subjected to separation into leaf and stem components, after which the samples comprising leaves and flowers were desiccated. Essential oil was subsequently extracted via water distillation utilizing a Neo-Clevenger apparatus for a duration of 2 h, resulting in a yield of 2.3%.

The collagen gel was prepared following the known technology previously described [53] and had 2.39% collagen (dry substance) and acidic pH (2–3). ZnO nanopowder (<50 nm particle size) and β-tricalcium phosphate (β-TCP) were purchased from Sigma-Aldrich. Sodium hydroxide (NaOH) and glutaraldehyde (GA) were from Merck (Frankfurt, Germany).

### 2.2. Preparation of Fillers

β-TCP with or without ZnO and collagen (1%) at a ratio of 1:2 was mixed and formed composite gel fillers. The obtained composites were adjusted to physiological pH using NaOH (0.1 M). ZnO was modified with different concentrations of *S. thymbra* L. EOs. The compositions and codes of composite gel fillers are presented in Table 1.

The spongious forms were obtained by freeze-drying of composite gels, as we previously described [54]. Briefly, the composite gel fillers were cast in glass Petri dishes with a diameter of 5.2 cm and a height of 1.0 cm, left for 24 h at 4 °C for crosslinking and then placed on the Delta 2–24 LSC freeze-dryer(Martin Christ Gefriertrocknungsanlagen GmbH, Osterode am Harz, Germany). The shelves of the freeze-dryer were previously cooled to −40 °C and then maintained for 6 h with the gel fillers at same temperature. The process continued with the main freeze-drying at 0.12 mbar and −40 °C. Then, the temperature increased to 10 °C in 10 h, 20 °C in 12 h and 30 °C in 12 h at the same pressure (0.12 mbar). The final freeze-drying took place in 2 steps: 3 h at 30 °C at 0.001 mbar and 3 h at 35 °C at 0.001 mbar. After this time, the bone fillers were obtained in spongious forms.

The spongious bone fillers L1–L6 were characterized and tested by physical–chemical, structural, morphological, and microbiological features as well as for biocompatibility with human BMSCs.

### 2.3. Water Uptake of Biocomposites

The spongious fillers with a size of about 1 cm^3^ were used to determine the water uptake capacity by the gravimetric method, as we previously described [55]. Briefly, the samples were weighed before and after immersion in water at different intervals of time, at room temperature (about 20 °C). The water uptake was measured using the following equation (Equation (1)):

*Water uptake* (%) = [(*Wt* − *Wd*)/*Wd*] × 100 (1)

where *Wt* represents the weight of water retained by sponges at time *t*, and *Wd* is the weight of dry sponges. Data were presented as mean ± standard deviation (SD) of three independent experiments.

### 2.4. The Biodegradation of Spongious Fillers into Collagenase Solution

The biodegradation capacity of biocomposite sponges was measured in vitro in collagenase solution (10^−6^ mg/mL) in phosphate saline buffer (PBS) at 7.4 pH. Each sponge uptook water to saturation, and then they were digested in collagenase solution (3 mL) at 37 °C in an oven. At specific time intervals, the sponges were weighed, and the weight loss was calculated using the following equation (Equation (2)):

*Weight loss* (%) = [(*Wo* − *Wt*)/*Wo*] × 100 (2)

where *Wo* represents the initial weight of sponges immersed in water, and *Wt* is the weight of sponges after the immersion in collagenase solution at time t. This experiment was performed in triplicate.

### 2.5. Fourier Transform Infrared Spectroscopy (FT-IR)

The FT-IR with ATR (Attenuated Total Reflectance) measurements of samples were carried out in ambient conditions using a Perkin–Elmer Spectrum Two IR spectrometer (PerkinElmer, Waltham, USA). All spectra were recorded in the wavenumber range between 400–4500 cm^−1^ at room temperature using a DTGS detector. Each ATR—FT-IR spectrum is the average over 10 scans, using air as reference and 2 cm^−1^ as nominal spectral resolution.

### 2.6. Scanning Electron Microscopy of Biocomposites

The morphology of biocomposite sponges was observed using the Hitachi SU 8230 Scanning Electron Microscope (Hitachi, Tokyo, Japan) equipped with an Oxford EDX detector–analyzer for sample analysis.

### 2.7. Mechanical Tests

Multiple pinched specimens (round-shaped, diameter of around 10 mm at a thickness of around 2 mm) were cut from each of the collagen-based lyophilized samples and use as compression samples. All testing was carried out using a computer control electromechanical universal testing machine (WDW-150, Jinan Scientific Test Technology Co., Ltd., Jinan, China) at room temperature. A 5 kN sensing cell was used to monitor the stress–strain relationship. The presented values are the average values calculated from five measurements.

### 2.8. Characterization by Microbiological Analysis

The disk diffusion assay was used to check the antibacterial activity of composite sponges against *Staphylococcus aureus* and *Escherichia coli* by measuring the diameter of the growth inhibitory zone. The samples were tested on ATCC strains from the collection of the ICPI Biotechnology Laboratory, namely, on *Escherichia coli* (ATCC 10536) and *Staphylococcus aureus* (ATCC 6538). A tube containing Mueller Hinton Broth (MHB) inoculated with *E. coli* and *S. aureus* was incubated for 24 h at 37 °C. Decimal dilutions up to 10^−5^ CFU/mL were made from this tube. A total of 200 μL of the inoculum was seeded onto the plates. Afterwards, the samples thus prepared were added and kept in the incubator for 24 h, at 37 °C. After this time, the antibacterial activity was determined by measuring the diameter of the zone of inhibition around the sample.

### 2.9. Cell Culture

Human BMSCs were isolated using a modified protocol established by our group [56], after informed consent, in accordance with the most recent version of the Helsinki Declaration of the World Medical Association (Ethical Principles for Medical Research Involving Human Subjects, October 2008). The cell isolation procedure was approved by the Institutional Ethical Committee (180 from 27 September 2018). Briefly, BMSCs were grown in DMEM 1 g/L glucose supplemented with 10% fetal bovine serum (FBS), 1% non-essential amino acids, 100 IU/mL penicillin, 100 µg/mL streptomycin, 50 µg/mL neomycin, at 37 °C and 5% CO_2_. The cells were characterized following the indications of the International Society for Cellular Transplantation [57], as our group showed previously [58]. For all the assays which were performed, the cells were seeded at the density of 1.5 × 10^5^ cells/cm^2^ on top of the 5 mm diameter composites. The tests were run in two independent experiments with four replicates for each condition, and the results are presented as mean ± SD.

### 2.10. Cell Viability Assay

The cell viability of human BMSCs seeded on the composites was assessed using the XTT (2,3-Bis-(2-methoxy-4-nitro5-sulfophenyl)-2H-tetrazolium-5-carboxanilide) method, according to the manufacturer’s instructions (Thermo Scientific, Waltham, MA, USA). A TECAN spectrophotometer (TECAN, Männedorf, Switzerland) was used to measure the absorbance at 450/650 nm, which is correlated with the quantity of viable and metabolically active cells. The results were reported as percentage of the control (L1 sample).

### 2.11. Cell Proliferation Assay

The cell proliferation was assessed by quantification of the nuclear DNA through fluorescence staining. Briefly, after the same interval of time (5 days) from seeding the cells, the sponge composites were briefly washed with PBS and the cell membranes were lysed by liquid nitrogen for nuclear DNA release. Then, the samples were incubated for 1 h with 10 μg/mL solution of nucleic acid dye Hoechst 33342 (Sigma-Aldrich, St. Louis, MO, USA). The fluorescence, which is proportional to the DNA concentration and to the number of the cells, was measured in black flat-bottom plates using the TECAN fluorescence plate reader (TECAN, Männedorf, Switzerland) at 350 nm excitation and 460 nm emission wavelengths. The recorded fluorescence values were converted to DNA quantities using a standard curve made with salmon DNA (Invitrogen, Carlsbad, CA, USA). The DNA content of the cells cultured on each condition was reported to the values of the control sample (L1) and showed as arbitrary units.

### 2.12. Assessment of Capacity to Support Colonization of Human BMSCs

Following the viability and proliferation assays, BMSCs were seeded on top of the composites placed in a 96-well plate. After 14 h, the samples were transferred to new wells to further cultivate only the BMSCs that adhered to the matrix. The medium was changed after two days, and on day five post-seeding, the composites were fixed in 4% PFA (paraformaldehyde) (Sigma Aldrich, St. Louis, MO, USA), overnight, at 4 °C, followed by inclusion in a Shandon Cryomatrix (Thermo Scientific, Waltham, MA, USA) and cryosectioning. To assess the cell colonization capacity of the sponge composites, we performed an eosin–Hoechst staining. Briefly, the sections were fixed with methanol, washed in PBS, followed by 2 min in eosin and differentiation in 70% ethanol and distilled water for 1 min, and the nuclei were stained with Hoechst 33342 solution (Sigma Aldrich, St. Louis, MO, USA) for 10 min. After a final wash and mounting with Shandon Immu-Mount (Thermo Scientific, Waltham, MA, USA), the samples were visualized using a Zeiss Observer D1 fluorescence microscope (Zeiss, Oberkochen, Germany).

### 2.13. Filamentous Actin (F-Actin) Staining of Cells

In order to visualize the filamentous actin (F-actin) of human BMSCs cultured on the sponge composites for 5 days, the cryosections were washed with PBS, fixed and permeabilized with 4% PFA (Sigma Aldrich, St. Louis, MO, USA) containing 0.1% Triton X-100 (Sigma Aldrich, St. Louis, MO, USA) and washed twice with PBS. The fluorescent Alexa 488-conjugated Phalloidin (A12379, Thermo Scientific, Waltham, MA, USA) was added for 1 h at room temperature in the dark, at concentration indicated by the manufacturer. In the end, the samples were washed three times with PBS and stained with Fluoroshield mounting solution with DAPI (Sigma Aldrich, St. Louis, MO, USA) to visualize the cell nuclei. Images were acquired with a Zeiss Observer D1 fluorescent microscope (Zeiss, Oberkochen, Germany).

### 2.14. Alkaline Phosphatase (ALP) Activity Assay

Alkaline phosphatase (ALP) activity was assessed on day 5, after seeding the human BMSCs on top of the samples, by measuring the transformation of p-nitrophenyl-phosphate (pNPP) into p-nitrophenol (pNP). Briefly, the cells were washed twice with warm PBS, and pNPP solution (the substrate for ALP) was added to each well. The plates were then incubated at 37 °C, 5% CO_2_ for 1 h. The amount of p-nitrophenol produced by the cells cultured on sample surfaces indicates the ALP activity level. After incubation, the supernatant was transferred to a 96-well plate for the colorimetric assessment. The absorbance was measured at 405 nm using a TECAN reader (TECAN, Männedorf, Switzerland), and the p-nitrophenol concentration was calculated by interpolation using a 20 μM to 200 μM pNP standard curve. Then, the amount of p-nitrophenol was normalized to the DNA content recorder in the proliferation assay, and the values for ALP activity were reported as nmols p-nitrophenol/ng DNA/min.

## 3. Results and Discussion

**Figure 1:**
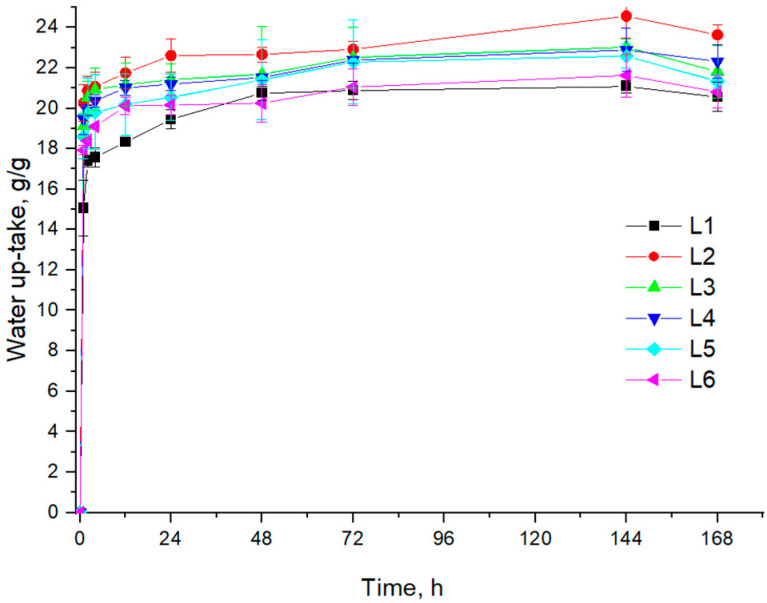
Water uptake of composite sponges L1–L6.

The capacity of composite sponges to swell in water is presented in Figure 1.

All the composites L1–L6 absorbed over 15 g/g water fast, in the first minutes, and then they swelled slowly in the first 24 h and reached equilibrium. The composite consisting of collagen and β-TCP (L1) absorbed the smallest amount of water, reaching about 20 g/g after 6 days. The adding of ZnO increased the water absorption; such results were also obtained by Andonegi et al. [59], when they characterized different concentrations of ZnO nanoparticles incorporated in native collagen films. All the samples based on collagen, β-TCP and ZnO absorbed 17–20 g/g water in the first minutes.

The water uptake ability decreased when the EOs were added, and this is the result of the hydrophobic feature of EOs.

A higher amount of *S. thymbra* EO resulted in less water absorption. However, the influence of EOs was not significant, with differences between samples ranging from 1 to 3 g/g. Starting from day 7, all samples began to degrade, showing a reduced water absorption rate.

We supposed that the water absorption is slightly decreased when the EO is added due to the hydrophobic character of EO. The collagen has a very high hydrophilic character, but is inhibited when the EOs are added.

**Figure 2:**
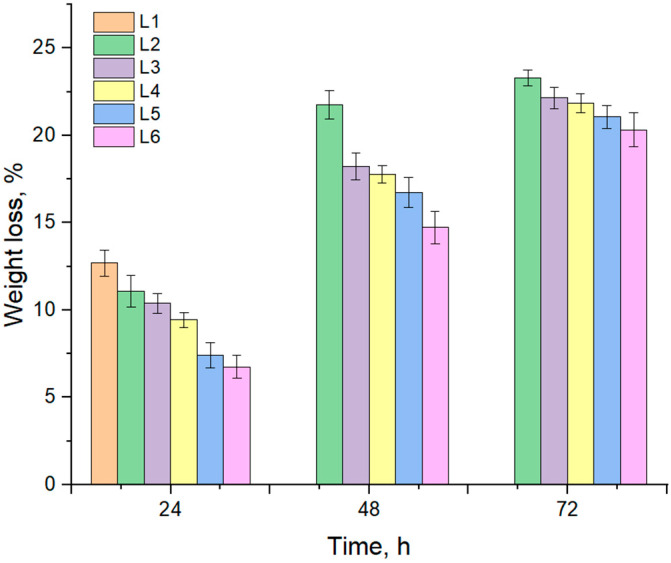
Biodegradation of composite sponges L1–L6.

After full swelling, the composite sponges were degraded in collagenase, a specific enzyme for collagen, which degrades the collagen molecules down to amino acids. Figure 2 presents the biodegradation of composite sponges L1–L6 during 72 h.

The sample L1, which does not contain ZnO, degraded by about 13% in 24 h, but could not resist more than 48 h, when it was totally degraded. The samples with ZnO degraded slowly, reaching about 7–11% in 24 h, 14–21% in 48 h and 20–23% in 72 h. It seems like the collagenase is inhibited by the action of components from *S. thymbra* EO; ZnO interaction with collagen can also be the reason for the slow degradation of collagen in simulated physiological conditions. Monfared-Hajishirkiaee et al. suggested in their research that ZnO NPs can slightly delay the biodegradation due to their cross-linking of Zn^2+^ ions to the sodium alginate [40,60]. After 72 h, the sponges did not degrade any further for weeks, the interaction between components being strong, blocking or inhibiting the collagenase solution to degrade the composites. This is a strong advantage for a bone filler which acts in the beginning as a treatment for a bone defect. It is known that secondary healing of the bone takes between 6–12 weeks and involves a specific biological pathway: hematoma formation, granulation tissue formation, callus formation, and bone remodeling. However, immediately after the inflammatory phase, the formation of the new callus begins and lasts only 5–10 days [61]. The biodegradation results sustain the idea of our study to provide a support on which cells could begin to synthesize extracellular matrix proteins, thus intervening in the initial stages of bone regeneration.

**Figure 3:**
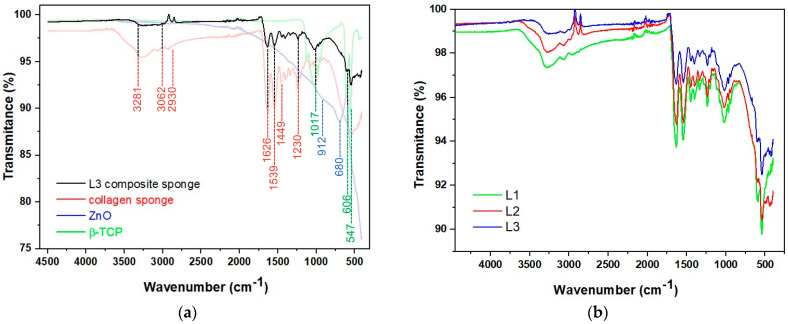
FT-IR spectra of (**a**) a sponge composite (L3) and its main components and (**b**) overlap of FT-IR spectra for L1, L2 and L3 composites.

The interaction between the components is given by FT-IR analyses as follows. Figure 3a presents the FT-IR for each component of composites, and Figure 3b shows the overlap of FT-IR spectra for composite sponges L1—collagen and β-TCP, L2—collagen, β-TCP and ZnO and L3—collagen, β-TCP and ZnO modified with *S. thymbra EO*. A similar pattern of the FT-IR was registered for composites L3–L6, proving that the small amount of essential oil (i.e., 25 nL reported to 1 g of collagen according to Table 1) does not influence the structure of samples. Because of this, only L3 overlapped with L1 and L2.

The vibration bands 3281 cm^−1^ (Amide A), 3062 and 2930 cm^−1^ (Amide B), 1626 cm^−1^ (Amide I), 1539 cm^−1^ (Amide II), 1230 cm^−1^ (Amide III) and 1449 cm^−1^ (pyrolidonic ring of hydroxyproline) correspond to the collagen structure [62,63,64].

The bands at 1118, 1017, 965, 942 as well as 606, 547 cm^−1^ are associated with the P-O bond of phosphate groups [36,65,66,67,68,69]. The band from 1328 cm^−1^ is not specific for collagen, being formed in the biocomposite as a result of interaction between the Ca^2+^ ions from CaP and collagen–COO groups due to mineral deposition [67,68]. When ZnO was added, the band from 1328 cm^−1^ shifted to 1338 cm^−1^ and the band from 1393 to 1400 cm^−1^; other changes of spectra were visible in Amide B, when peaks shifted from 2946 cm^−1^ to 2977 cm^−1^ and, in Amide III, from 1233 to 1239 cm^−1^. These changes can be due to interaction of collagen with minerals (β-TCP and ZnO) during composite formation.

**Figure 4:**
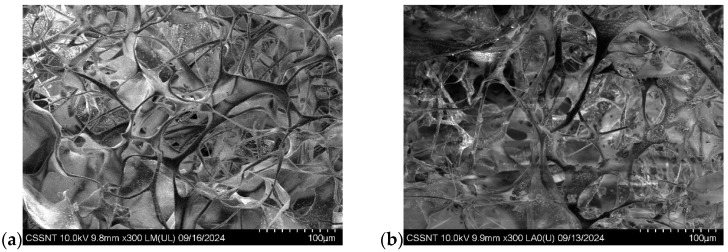

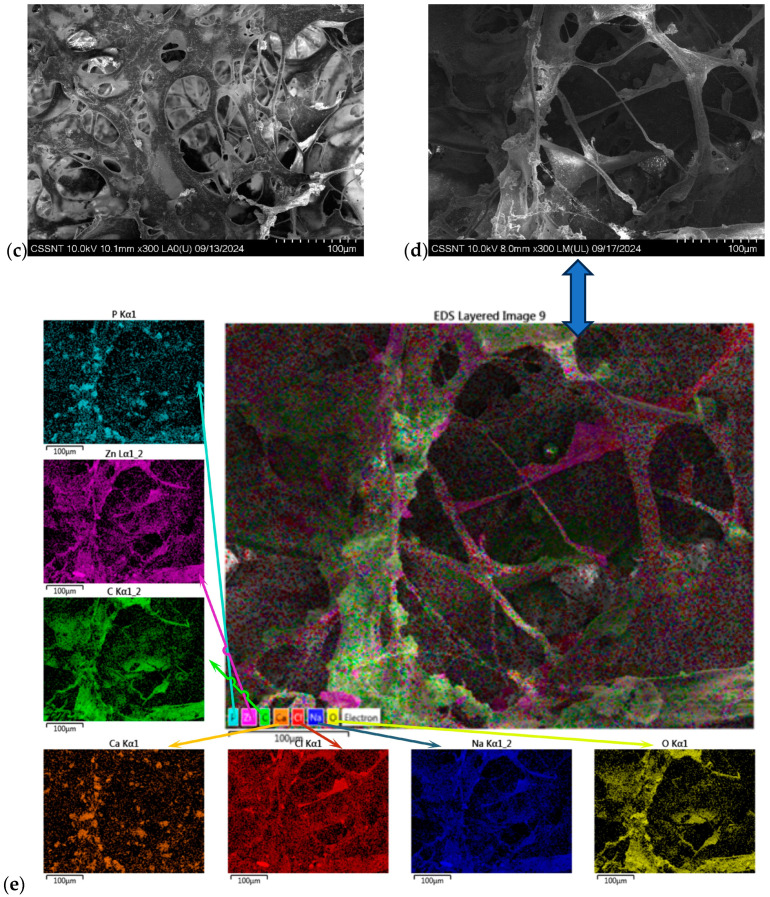
SEM of sponge composites: (**a**) collagen control; (**b**) L1; (**c**) L2; (**d**) L6; (**e**) EDX map of L6 sponge composite (magnification ×300).

The morphology of main spongious composites is presented in Figure 4a–d.

All the composites showed a porous structure with interconnected pores with pore sizes between 50–150 μm, the proper sizes for bone regeneration facilitating cell infiltration and proliferation of osteoblasts and mesenchymal cells, nutrient flow, and vascularization [70,71]. The collagen fibrils are very evident in each sample, and the interaction between collagen and minerals (β-TCP and ZnO) did not affect the 3D structural arrangement. An exemplification of the sample L6 using EDX at ×300 magnification (Figure 4e) showed the accumulation of calcium, zinc, and phosphate on the collagen fibrils. The Na and Cl ions come from the technological process of obtaining collagen. It is visible that the components are very homogeneously spread into the sponge.

Table 2 shows the average of Ca ions from β-TCP and Zn ions from ZnO as a percentage of weight and the corresponding standard deviation (SD).

From Table 2, it is visible that the L1 sample, which does not consist of ZnO, has a very small amount of Zn, which can be from collagen synthesis (skin being the third most Zn-abundant tissue in the body), and all the other samples consist of very similar amounts of Zn, varying from 6.28 to 7.01%. The amount of Ca is also very similar in all the samples.

**Figure 5:**
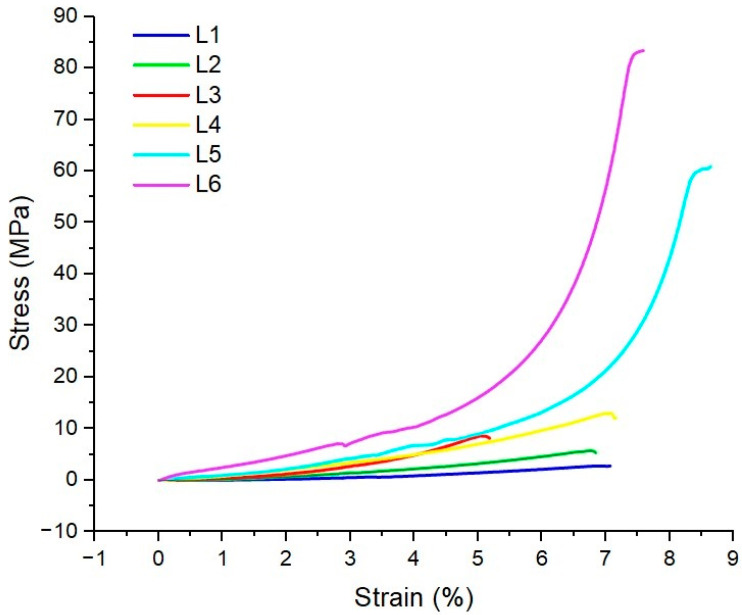
Compressive stress–strain curve of L1–L6 sponge composites.

Compressive stress–strain curves of L1–L6 composite fillers are presented in Figure 5.

The mechanical properties of the sponge composites are influenced by the porosity and the structure of materials [2,72]. As we can see in Figure 5, sample L1 without ZnO decreased strain and stress compared with L2–L6. The difference between samples with the same amounts of ZnO and different concentrations of essential oils is also visible, the mechanical properties of samples being improved when *S. thymbra* EO concentrations increased. The results are in correlation with biodegradation tests and water absorption. The antibacterial activity is very important both before and during implantation of bone fillers, an infection resulting in strong complications of surgical actions and even death. The antibacterial activity of ZnO, its beneficial effects in the various stages of the wound-healing process but also the cytotoxic effects above 2% [39,73,74] are well known. Our unpublished results showed that even a reported concentration of 1% of ZnO results in cytotoxic effects. Sasan et al. found a synergy between ZnO with *Salvia abrotanoides* EOs into Polycaprolactone (PCL)/alginate core-shell nanofibers, proving antibacterial and anti-inflammatory properties [74].

**Figure 6:**
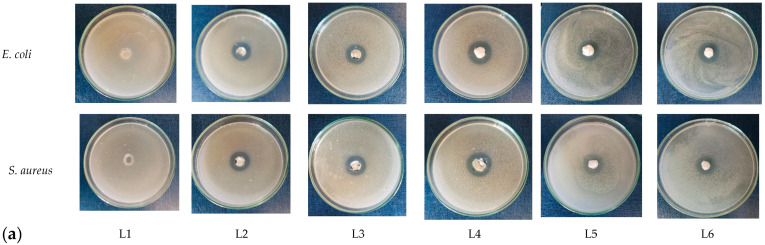

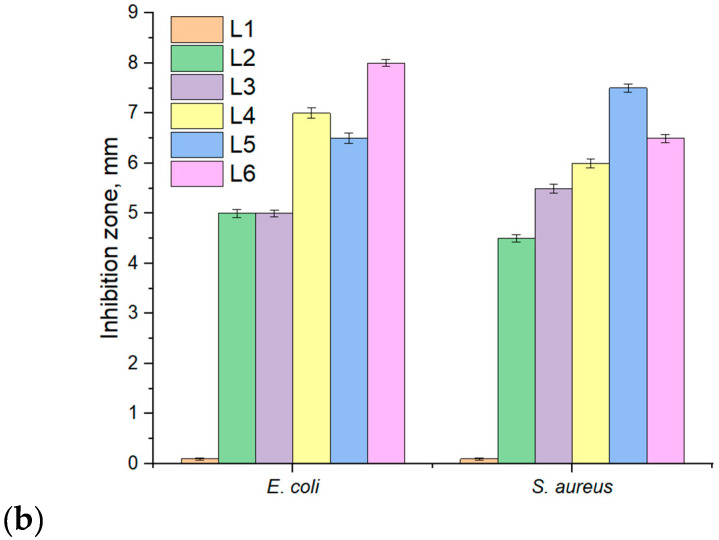
Antibacterial activity of sponge composites L1–L6 against *E.coli* and *S. aureus* measured by disc diffusion method: (**a**) images of samples and (**b**) size of inhibition zone, mm.

We choose *S. thymbra* L. essential oil to improve the biological properties of bone filler. Figure 6a,b present the behavior of sponge composites (L1–L6) against *S. aureus* and *E. coli*.

As Figure 6 shows, there is no antibacterial activity for sample L1, which does not contain ZnO and EO. The antibacterial activity was present for all the samples with ZnO content, and the inhibition zone started to increase when the *S. thymbra* L. EO was added. The L6 sample showed the highest antibacterial activity against *E. coli*, and L5 showed the same against *S. aureus*. This indicates that the combination of ZnO and *S. thymbra* L. EO has a synergic effect.

**Figure 7:**
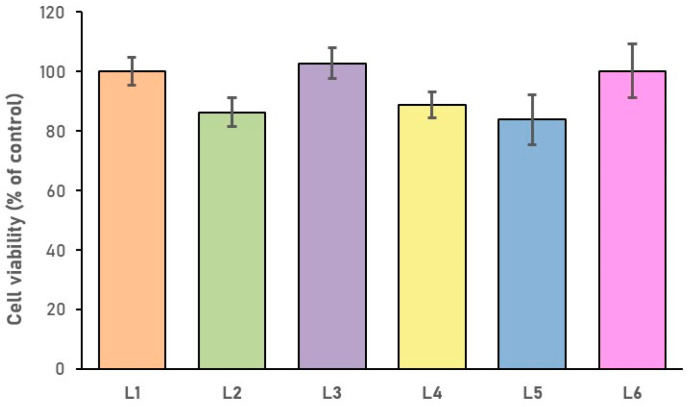
Evaluation of cytotoxic effect of the sponge composites using the XTT assay. The viability of human BMSCs cultured for 5 days on the samples was assessed. Data represent mean ± SD, expressed as percentage of the control viability (L1 sample).

The cytotoxicity of the sponge composites was assessed using the XTT test. The results presented in Figure 7 indicate that no cytotoxic effects against human BMSCs were observed. In all the tested samples, the viability is comparable to that of the control (L1 sample), with values exceeding 83.7% ± 8.5 (L5 sample).

**Figure 8:**
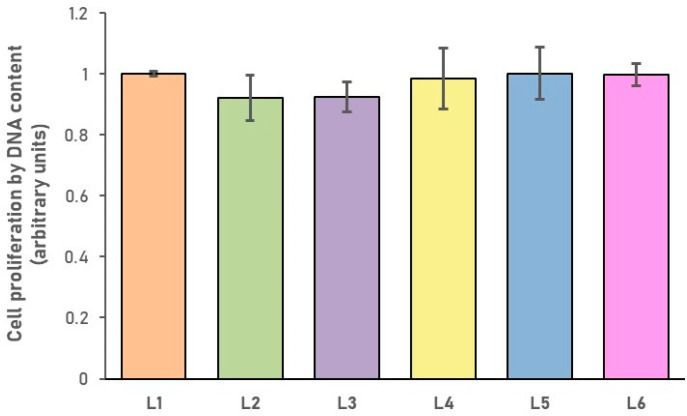
Determination of the capacity of the sponge composites to sustain human BMSC proliferation. Data represent mean ± SD of the DNA content, expressed as arbitrary units.

The results of the cell proliferation assay are represented in Figure 8, showing the proliferative behavior of human BMSCs seeded on top of the scaffolds. The DNA content of the cells grown under all conditions is comparable to the amount of DNA of the cells cultured on the control sample (L1), showing that the different concentrations of *S. thymbra* L. EO did not affect the cell proliferation.

**Figure 9:**
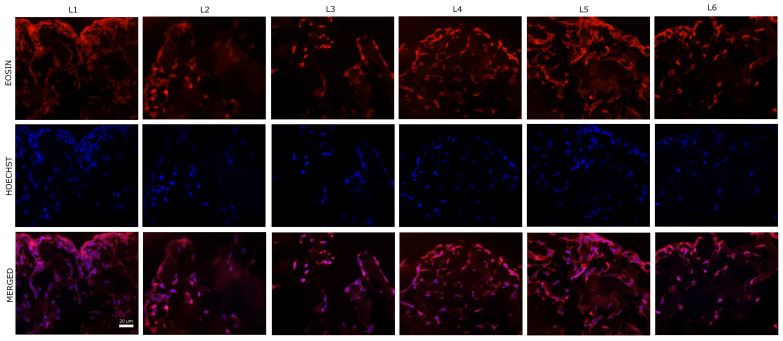
Representative images of eosin-Hoechst staining of the sponge composites seeded with human BMSCs (5 days post-seeding)—peripheral region of the composites (40× magnification). Nuclei stained with Hoechst (blue) and cells cytoplasm stained with eosin (red).

Regarding the colonization capacity of the sponge composites, the eosin–Hoechst staining revealed that human BMSCs adhered well, showing a thick and spread-out distribution of cells across the seeded surface. Cell spreading is an essential function of the cells which have adhered to a surface and precedes the function of cell proliferation, resulting in a fully cell-covered surface [75,76]. Representative images from the peripheral (seeded) surface of the scaffolds are presented in Figure 9.

**Figure 10:**
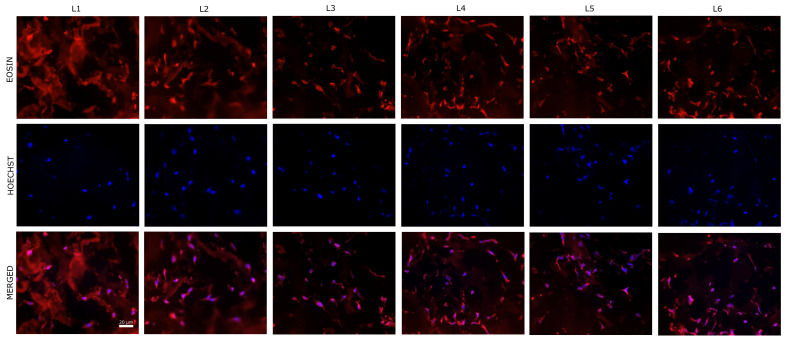
Representative images of eosin-Hoechst staining of the sponge composites seeded with human BMSCs (5 days post-seeding)—central region of the composites (40× magnification). Nuclei stained with Hoechst (blue) and cells cytoplasm stained with eosin (red).

Moreover, the cells migrated into the composites, the cell density being a bit lower in the center. Representative images from the central zone of the scaffolds are presented in Figure 10.

**Figure 11:**
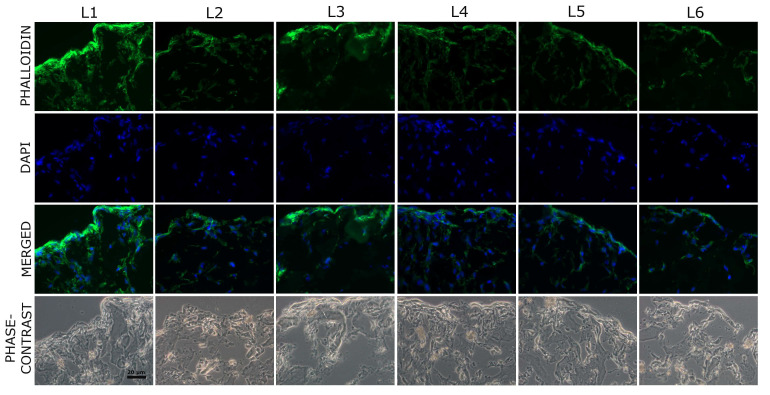
Representative images of human BMSCs stained for F-actin on sponge scaffolds after 5 days of culture—peripheral region of the composites. Fluorescent images of F-actin (green), nuclei (blue), merged images and phase contrast images are shown. Magnification: 40×.

Cell adhesion is a complex process involving the reorganization of cytoskeleton proteins like actin. Thus, F-actin staining was used to examine the cell behavior on day 5 post-seeding. Precisely, it helped in visualization of the cell attachment and distribution on the scaffolds. As shown in Figure 11, the upper surfaces (seeding surface) are densely populated, being almost completely covered by a cell layer. At this level, the morphology of human BMSCs is highly elongated. In addition, the cells were found to spread to the central regions of the scaffolds, maintaining their characteristic spindle shape. Phase contrast light microscopy images of the same areas reveal the porous structure of the scaffold, indicating the cell adherence.

**Figure 12:**
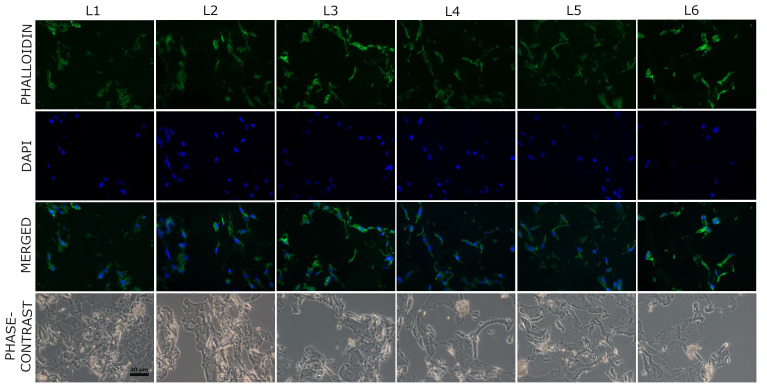
Representative images of human BMSCs stained for F-actin on sponge scaffolds after 5 days of culture—central region of the composites. Fluorescent images of F-actin (green), nuclei (blue), merged images and phase contrast images are shown. Magnification: 40×.

As we can see in Figure 12, the used porosity is suitable for cell migration inside the scaffolds. It is well known that the pore network is important in order to obtain a relatively homogenous colonization [77]. In the central regions of the scaffolds, the cells presented the same morphology being distributed across the collagen fibers (Figure 12).

**Figure 13:**
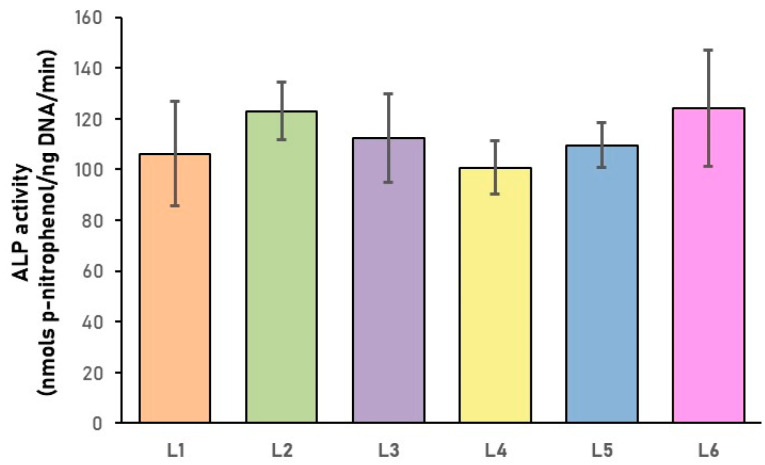
ALP activity of human BMSCs seeded on the sponge composites. ALP activity was measured after 5 days of culture and normalized by DNA content of the cells. Values reported as mean ± SD represent nmols p-nitrophenol/ng DNA/min.

These results reveal that the pore size (50–150 µm) allowed in the first step the adhesion and proliferation of BMSC on all tested scaffolds, as shown in Figure 8 and Figure 9. Moreover, as seen in Figure 10, after 5 days in culture, cells were able to migrate and colonize the whole scaffold, entering and proliferating into the inner regions. This demonstrates the nutrient diffusion into all regions of the scaffolds. The ALP enzyme has a critical function in the formation of hard tissue, being highly expressed in the cells of mineralized bone. It is produced early in growth and is easily found on the surface of the cells and in vesicles from the matrix in calcifying tissues [78]. ALP activity levels present in the human BMSCs after 5 days of seeding on the sponge composites are similar to the control (L1 sample) for all the tested conditions (Figure 13).

These results suggest that *Satureja thymbra* EO does not impede the mineralization process, which makes the tested biomaterials suited for bone implants supporting tissue regeneration.

## 4. Limitations of the Study

While this study demonstrates the cytocompatibility and structural stability of the tested scaffolds over a 5-day period in the presence of human BMSCs, certain limitations must be acknowledged. First, although no degradation was observed within the tested timeframe, longer-term studies are necessary to confirm the stability and biocompatibility of the scaffolds over extended periods. Moreover, the study focuses exclusively on in vitro conditions, which may not fully reproduce the complexity of the in vivo environment. Factors such as interactions with various cell types, immune responses, and the dynamic mechanical forces present in living tissue could influence the efficacy of the biomaterials. However, the results presented in this study are promising for promoting bone regeneration and provide a foundation for future research to explore the biomaterial behavior in in vivo condition.

## 5. Conclusions

This research aimed to develop new sponge composites as bone filler as antibacterial implants for bone defects. The composition of sponge composites consists of collagen and β-TCP and also an antibacterial agent such as ZnO and ZnO modified with *S. thymbra* L. EOs. In order to characterize the spongious composites, FT-IR, SEM with EDX, water uptake, and biodegradation analysis were used, and they showed suitable properties for a bone filler, such as high porosity with interconnected pores, homogeneous composition, high absorbance, unaltered collagen structure, and lower biodegradability; the antibacterial activity was evaluated against *E. coli* and *S. aureus* and showed good properties when ZnO is present in the composition and a synergic effect when EO is added. They are also biocompatible with human BMSCs, with sustained adherence, proliferation and colonization. These findings reveal that the sponge composites are both biocompatible and antibacterial at the same time. Moreover, ALP activity in human BMSCs grown on these scaffolds can be predictors for bone mineralization. Demonstrating the balance between properties, the samples L5 and L6 can be good candidates as bone fillers for implantation.

## Figures and Tables

**Table 1:** Composition of fillers.

Codes of Fillers	Collagen *, %	β-TCP **, %	ZnO **, %	*S. thymbra* EO *, *%*	GA *, %
L1	1	0.5	0	0	0.25
L2	1	0.45	0.5	0	0.25
L3	1	0.45	0.5	0.0025	0.25
L4	1	0.45	0.5	0.005	0.25
L5	1	0.45	0.5	0.025	0.25
L6	1	0.45	0.5	0.05	0.25

* reported to gel amount. ** reported to collagen, dried substance.

**Table 2:** Average of Ca and Zn ions from all fillers through EDX analysis.

Codes of Fillers	Average of Ca wt., %	SD	Average of Zn wt., %Zn	SD
L1	7.16	2.37	0.39	0.78
L2	7.92	1.17	6.75	0.51
L3	8.07	3.04	6.92	1.41
L4	7.66	1.02	7.01	2.38
L5	7.49	3.67	6.99	1.21
L6	7.93	1.41	6.28	0.47

## Data Availability

The original contributions presented in the study are included in the article, further inquiries can be directed to the corresponding author.
